# Institutionalizing Provider-Initiated HIV Testing and Counselling for Children: An Observational Case Study from Zambia

**DOI:** 10.1371/journal.pone.0029656

**Published:** 2012-04-20

**Authors:** Jane N. Mutanga, Juliette Raymond, Megan S. Towle, Simon Mutembo, Robert Captain Fubisha, Frank Lule, Lulu Muhe

**Affiliations:** 1 Livingstone General Hospital, Livingstone, Zambia; 2 Albuquerque, New Mexico, United States of America; 3 Mumbai, India; 4 Southern Provincial Medical Office, Livingstone, Zambia; 5 Department of Paediatrics and Child Health, Livingstone Paediatric Centre of Excellence, Livingstone, Zambia; 6 World Health Organization Regional Office for Africa, Brazzaville, Congo; 7 World Health Organization Headquarters, Geneva, Switzerland; UCL Institute of Child Health, University College London, United Kingdom

## Abstract

**Background:**

Provider-initiated testing and counselling (PITC) is a priority strategy for increasing access for HIV-exposed children to prevention measures, and infected children to treatment and care interventions. This article examines efforts to scale-up paediatric PITC at a second-level hospital located in Zambia’s Southern Province, and serving a catchment area of 1.2 million people.

**Methods and Principal Findings:**

Our retrospective case study examined best practices and enabling factors for rapid institutionalization of PITC in Livingstone General Hospital. Methods included clinical observations, key informant interviews with programme management, and a desk review of hospital management information systems (HMIS) uptake data following the introduction of PITC. After PITC roll-out, the hospital experienced considerably higher testing uptake. In a 36-month period following PITC institutionalization, of total inpatient children eligible for PITC (n = 5074), 98.5% of children were counselled, and 98.2% were tested. Of children tested (n = 4983), 15.5% were determined HIV-infected; 77.6% of these results were determined by DNA polymerase chain reaction (PCR) testing in children under the age of 18 months. Of children identified as HIV-infected in the hospital’s inpatient and outpatient departments (n = 1342), 99.3% were enrolled in HIV care, including initiation on co-trimoxazole prophylaxis. A number of good operational practices and enabling factors in the Livingstone General Hospital experience can inform rapid PITC institutionalization for inpatient and outpatient children. These include the placement of full-time nurse counsellors at key areas of paediatric intake, who interface with patients immediately and conduct testing and counselling. They are reinforced through task-shifting to peer counsellors in the wards. Nurse counsellor capacity to draw specimen for DNA PCR for children under 18 months has significantly enhanced early infant diagnosis. The hospital’s bolstered antiretroviral supply chain, package of on-site HIV services, and follow-up care for children and families improved the continuum of service uptake.

**Conclusions and Significance:**

The clinical impact and operational experience emphasizes that institutional PITC is a feasible strategy for increasing access to paediatric HIV care, particularly in generalized epidemic settings.

## Introduction

Of the estimated 33.3 million people globally living with HIV/AIDS in 2009, 2.5 million were children under 15 years of age. An estimated 370,000 children were born with HIV in 2009, and over 90% of these new infections are in children living in sub-Saharan Africa [1]. Despite considerable global efforts to scale-up HIV prevention, care, and treatment, services for HIV-exposed and infected infants and children have lagged behind. Of Zambia’s population of 12 million, an estimated 1.1 million are living with HIV [2]. Despite significant scale-up of prevention of mother-to-child HIV transmission (PMTCT) programming in the country, approximately 120,000 children aged zero to 14 years are living with HIV, and 28,000 infants are infected with HIV annually [2]–[3]. An estimated 59,000 children in Zambia were in need of antiretroviral therapy (ART) in 2009, while national paediatric ART coverage was 36% [4].

Though increasing numbers of HIV-infected women are identified during pregnancy, and coverage of prevention of mother-to-child has improved–internationally 53% of pregnant women received antiretrovirals to reduce transmission in 2009, up from 9% in 2004–many children remain unidentified in the postnatal period [4]. There are significant challenges to scale-up of paediatric counselling, testing, and care. These include poor linkages from PMTCT programmes and the subsequent missed opportunities for testing during postnatal and child health care, challenges of providing virological testing required for early infant diagnosis (e.g. RNA or DNA polymerase chain reaction, or PCR), limited paediatric expertise amongst healthcare providers, and the prioritization of adult treatment and subsequent lag in availability of paediatric ART dosages [5]–[11].

When opportunities to test children are missed, exposed and infected children with unknown status do not receive critical care interventions like co-trimoxazole prophylaxis, ART, isoniazid preventive therapy, and other support [6], [12]. Widely used volunteer testing and counselling (VCT) and risk-based testing strategies are not suitable for preventing HIV-related mortality in children due to rapid disease progression and overlapping symptoms with other illnesses [13]–[15]. This is particularly so in countries with generalized HIV epidemics. In low resource settings, late HIV diagnosis and delayed care interventions result in high mortality among HIV-infected children [13]. Without any interventions, 50% of HIV-infected children die before their second birthday, and 75% die by age five [14].

The World Health Organization (WHO) and Joint United Nations Programme on HIV/AIDS (UNAIDS) emphasize the need to increase knowledge of HIV status–and thereby expand access to HIV interventions–by scale-up of provider-initiated testing and counselling (PITC). The WHO recommends that PITC be offered to all persons seen in health facilities within a generalized HIV epidemic, while ensuring confidentiality and informed consent. Once PITC is institutionalized, all health care providers should recommend HIV counselling and testing as a standard component of medical care to persons attending health facilities [13]. A package of HIV prevention, care, treatment, and support services should accompany PITC [5]. By the end of 2009, over two thirds of countries in sub-Saharan Africa, Latin America, and the Caribbean had introduced PITC policies [4].

Provider-initiated testing and counselling (PITC) is a priority strategy for paediatric HIV care. It should be used to determine HIV-infected women for PMTCT interventions, identify HIV-exposed infants for prophylaxis measures, and diagnose HIV-infected infants and children for initiating antiretroviral therapy (ART) and care interventions [16]–[17]. PITC has identified significant numbers of children living with HIV in inpatient settings in Zambia [18] and Uganda [19], inpatient and outpatient settings in Malawi [10], [19]–[21], and outpatient clinics in Zambia [22] and Cameroon [23]. Implementation models of routine inpatient PITC for children underscore that they are operationally feasible, incurring modest financial and human resource demands but capturing significant numbers of HIV-exposed and infected children for HIV care and treatment [10], [19]–[21].

This paper examines the paediatric uptake of counselling, testing, and HIV care after PITC was introduced at a second-level hospital in Zambia. In the nine months prior to the introduction of PITC at Livingstone General Hospital, only 40.8% of total admitted children eligible for counselling (n = 1632) accepted HIV testing. In a 36-month period following the introduction of PITC, of total inpatient children eligible for PITC (n = 5074), 98.5% of children were counselled, and 98.2% were tested.

This paper argues that institutionalized PITC is a feasible strategy for increasing access to paediatric HIV care, particularly in generalized epidemic settings. The high HIV prevalence in tested infants and children emphasizes the critical and urgent need for decentralized early infant diagnosis (EID) capacity in generalized epidemic settings. By examining the best practices and enabling factors that contributed to this rapid institutionalization of PITC, this case seeks to contribute to a wider discussion on increasing access to high-quality HIV prevention, treatment, and care services for children in resource-limited settings. The dissemination of experiences in translating policy into practice aims to benefit policy makers, programme managers, and implementing healthcare providers.

### Setting

Livingstone District in Zambia’s Southern Province has an HIV prevalence of 30.9%, more than double the national prevalence of 14.3%. While 90% of the province’s population reports that they know where to access HIV counselling and testing, only about 20% has undergone counselling and testing [24]. Livingstone General Hospital (LGH) is a second-level referral hospital for the province, serving a population of 1.2 million people [25].

In 2003, LGH began offering free antiretroviral therapy in line with national policy. Adult services scaled up faster than paediatric care. By 2006, the hospital had over 2,000 adults on ART and only 40 paediatric clients; this was far below the paediatric ART target for 10% of the adult population on ART. LGH was offering Voluntary Counselling and Testing (VCT) and Diagnostic Counselling and Testing (DCT) at the chest clinic or for suspected cases in the inpatient department. The uptake of testing was very low under these two strategies. Counselling and testing uptake was particularly low in the paediatrics department.

In 2006, LGH partnered with Columbia University’s International Center for AIDS treatment programs (ICAP) to establish the Livingstone Paediatric Centre of Excellence (LPCOE). The partnership sought to streamline services and optimize paediatric care, including: (a) restructuring the hospital’s physical space to address challenges in patient flow, (b) standardizing medical records between in- and outpatient HIV care for children, and (c) developing stronger mechanisms to provide comprehensive HIV care to families. In 2007 the Zambian Ministry of Health (MOH) circulated a National PITC Policy and Implementation Guidelines for Routine HIV testing in facilities where there is access to treatment and care, and conducted a nationwide assessment of pilot PITC programmes in order to report on implementation successes from a number of facilities [26]–[28]. While not specific to paediatric testing, the national PITC guidelines provided an impetus for planning, sensitization, and implementation at the hospital. During this same time, national efforts to scale-up PMTCT increased the numbers of patients seeking services, an opportune time to initiate PITC. Zambia has released paediatric guidelines for HIV testing and counselling in 2011, and currently uses the 2010 edition of the WHO guidelines for PITC.

## Methods

**Figure 1:**
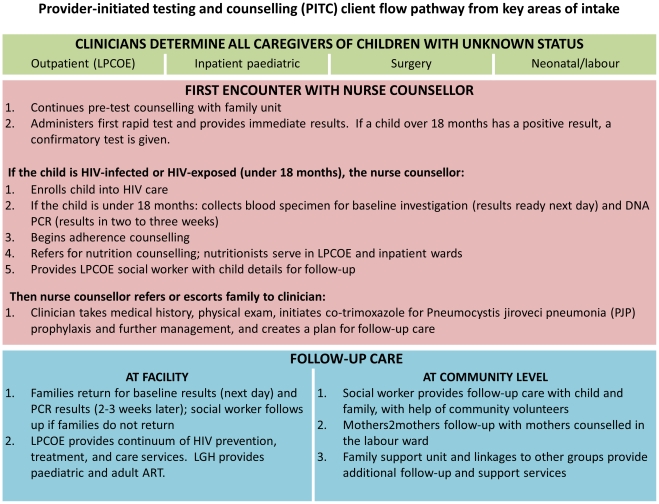
Provider-initiated testing and counselling (PITC) client flow pathway from key areas of intake. This figure charts the client flow pathway and activities involved from (a) four key areas of intake, where caregivers receive counselling, to (b) first encounter with nurse counsellor, which includes a number of described care practices, to (c) follow-up care at the facility and community level.

At the introduction of PITC, provider and patient flows were redesigned so that all eligible children can receive testing and counselling at their point of intake (Figure 1). Full-time nurse counsellors are stationed in the key areas of paediatric intake: the general paediatric and isolation wards, the admission ward, the outpatient clinic (this is the LPCOE for children), the surgical ward, and the neonatal and labour wards. The recently established Child Sexual Abuse One Stop Centre is an additional entry point to counselling and testing. All attending clinicians are trained to provide initial HIV counselling at each patient encounter. The nurse counsellor then attends to all children in the ward that are eligible for PITC, that is, those whose HIV status was not known or could be verified at the time of admission and/or have never had an HIV test before. The nurse counsellor then conducts pre-test counselling with the caregivers, or an individual session with an older child if the caregiver deems the child to be of appropriate age and maturity to discuss testing. All children under the age of sixteen require informed consent from a guardian. Relatives other than the biological parents are not in a position to give consent for testing, unless the child is orphaned. In the Zambian context, caregivers are most often female, and usually the biological mother, grandmother, aunt, or sometimes elder sister. At the same time, women are not usually in a position to makes decisions concerning the children for which they are caring, and will often request to consult the child’s father or grandfather before testing can be done. Children under the age of sixteen require guardian consent; children under this age who are married, pregnant, or parents are considered ‘emancipated minors.’

All children are provided a rapid diagnostic test (Abbot Determine). If a child over 18 months has a positive result in the first test, they are given a confirmatory test (Uni-Gold Recombigen) and tie-breaker if results are indeterminate (SD-Bioline). For children confirmed as HIV-infected, the nurse counsellors conduct post-test counselling, collect blood specimens for baseline investigations, and enrol clients into care before referring or escorting them to consultation with a clinician. If a child under 18 months has a positive result from their first rapid diagnostic test (Abbott Determine), the nurse counsellor immediately collects a dry blood spot (DBS) specimen for PCR testing. Midwives collect DBS from neonates in the labour ward in the absence of a nurse counsellor. DBS are sent by EMS courier for DNA PCR at the University Teaching Hospital’s central laboratory in Lusaka. When the results are returned in two to three weeks, caregivers are immediately contacted by phone. The LPCOE social worker follows-up with those who do not return for their tests, or cannot be reached by phone. When the caregiver and patient return to the hospital, they are provided post-test counseling, lab tests, further management plans, and linkages to social support networks if this has not already been done.

### Documentation

Documentation efforts examined retrospectively how the hospital and its partners utilized key scale-up strategies towards institutionalizing PITC and scaling up the accompanying paediatric HIV prevention, treatment, care, and support services. First, a desk study examined hospital HMIS data from 6706 children offered HIV testing during 48 months. This data was entered into excel and analysed by pre- and post-PITC periods using simple frequency distributions. As we only analysed aggregate, anonymous hospital administrative data, no ethics review was required. One limitation to note from the HMIS data is that the hospital began tracking children ‘eligible’ for PITC in January 2008, three months after PITC was introduced, thereby immediate pre- and post- service uptake cannot be tracked with specificity to eligibility. Second, a desk review of policy documents, technical guidelines, relevant memos or circulars, data collection tools used in national testing, and early infant diagnosis and procurement data. Third, hospital-based observations and key informant questionnaires administered to counsellors and hospital management examined operational successes and challenges.

## Results

First, this results section will examine both the operational results of PITC introduction, namely the significant increase in testing uptake and enrolment of HIV-infected children into care. Second, the section will review the operational best practices that permitted the increase in service delivery and uptake, as determined during key informant interviews and observations.

### Service Delivery Outcomes

#### Uptake of counselling and testing

**Figure 2:**
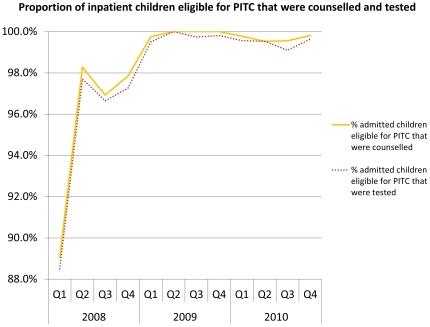
Proportion of inpatient children eligible for PITC that were counselled and tested. Line graph demonstrates the proportion of inpatient children eligible for PITC that (a) were counselled, and (b) were tested for HIV, by each quarter in the 36-month period following the introduction of PITC. Counselling and testing rates climb to over 99%.

Before PITC was introduced, there was low uptake of HIV testing in the department of paediatrics. In the nine months leading up to the October 2007 introduction of PITC, of all admitted children (n = 1632), 40.8% were tested for HIV. Routine PITC resulted in a dramatic shift (Figure 2). In a 36-month period following the introduction of PITC, of total inpatient children eligible for PITC (n = 5074), 98.5% of children were counselled, and 98.2% were tested. It is necessary to note that some admitted children were not eligible for counselling and testing during this period (n = 1746). This was most often because they already knew their status and the result could be verified, or the child arrived in an acute condition that required emergency resuscitation, or died before testing could be provided. Of the eligible children that were not tested, the most common reason was mothers wanted the consent of male partners, and never returned for their test.

#### Decentralized capacity enabling early infant diagnosis (EID)

**Figure 3:**
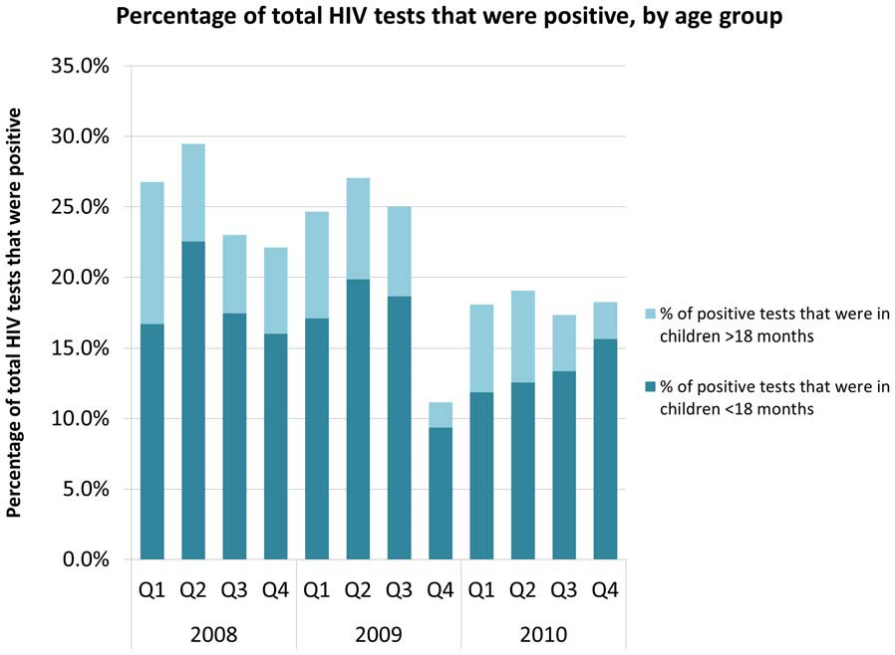
Percentage of total HIV tests that were positive, by age group. Bar graph demonstrates, by quarter in a 36-month period (a) what proportion of total inpatient and outpatient children tested were identified HIV-infected, (b) what proportion of these children were under and over 18 months old.

**Figure 4:**
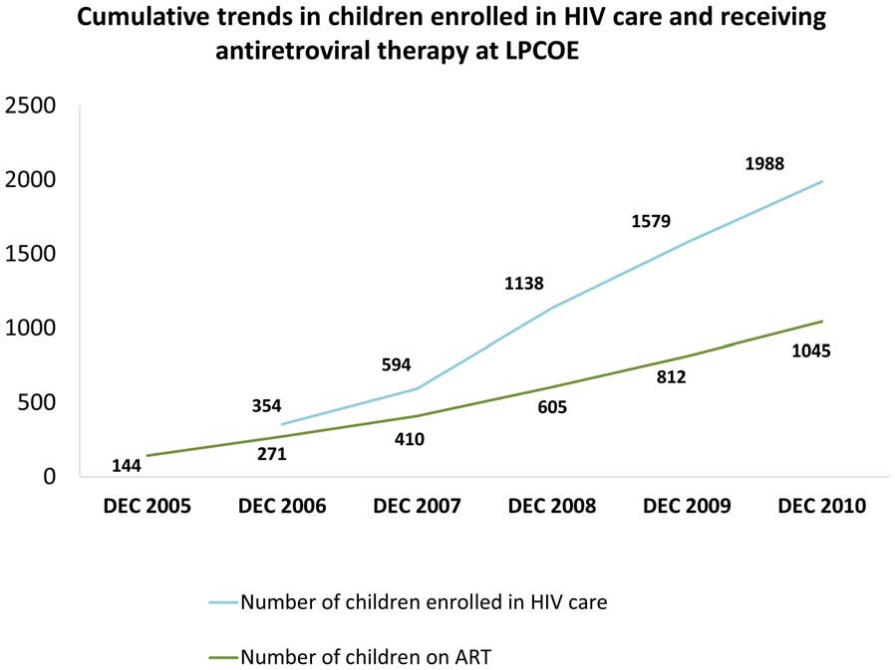
DNA PCR test results for children under 18 months in 2008 and 2009. This bar graph demonstrates the proportion of DNA PCR tests in 2008 and 2009 that were (a) disregarded due to poor collection or specimen management, (b) HIV positive, and (c) HIV negative. The figure is used to demonstrate how improved laboratory practices reduced the rate of disregarded tests between 2008 and 2009.

PITC also enabled the hospital to provide testing services for early infant diagnosis (EID) at the first encounter with an exposed child under 18 months in the outpatient clinics and on the wards. The mean age of children tested by PCR was 23 weeks old, and ages ranged from one day old to 18 months. In the 36 months after PCR was introduced within the PITC strategy, the method identified 72.7% of all children determined to be HIV-infected at testing in inpatient and outpatient settings (n = 1342) (Figure 3). The number of collected specimens increased rapidly with the introduction of PITC and scale-up of PMTCT services in the hospital’s catchment area. When early analysis of PCR results highlighted the need for quality control in collection, streamlined management and additional training, oversight, and quality control measures in the hospital laboratory were introduced. This saw a significant reduction in 2009 of the number of specimens disregarded due to collection more than three months prior (Figure 4).

#### Uptake into HIV care

**Figure 5:**
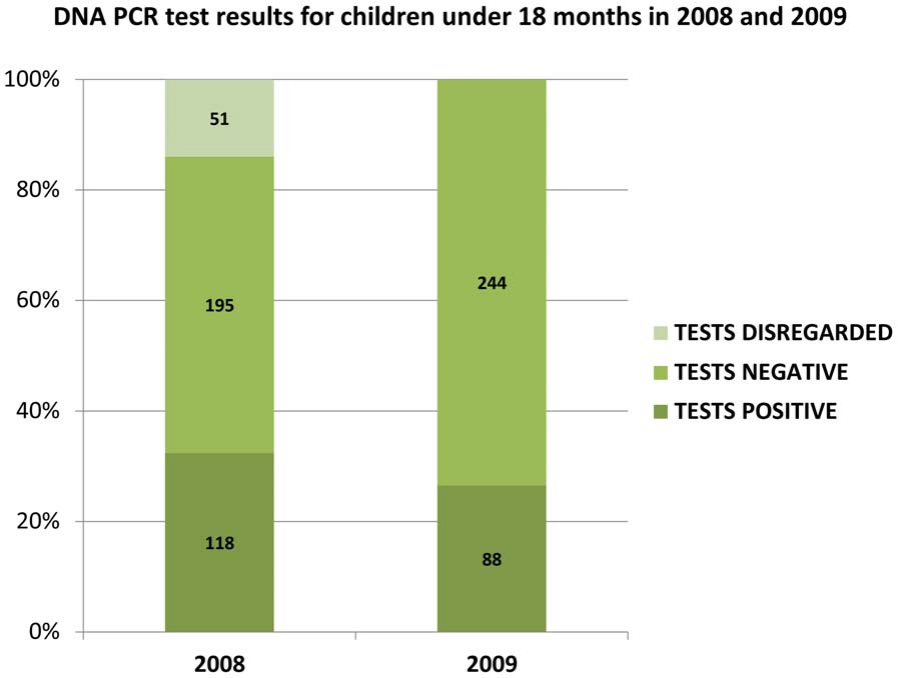
Cumulative trends in children enrolled in HIV care and receiving antiretroviral therapy at LPCOE. Line graph demonstrates the cumulative growth, reported annually from 2005–2009, of children (a) enrolled in HIV care at the facility, and (b) on antiretroviral treatment at the facility.

Of the children tested for HIV in inpatient and outpatient facilities between 2008 and 2010 (n = 6217), 21.6% were determined HIV-infected. This highlights a significant prevalence rate among children who have been missed in previous contacts with health workers, including postnatal follow-up of PMTCT, and reinforces the importance of offering PITC in all paediatric services in order to reach children who have slipped though the service net or were lost to follow-up. Of children identified as HIV-infected in the hospital’s inpatient and outpatient departments since 2008 (n = 1332), 99.3% were enrolled in HIV care, including initiation of co-trimoxazole for *Pneumocystis jiroveci* pneumonia (PJP) prophylaxis and other interventions. At the end of 2010, a cumulative total of 1,988 children were enrolled in LPCOE, and 883 children were receiving ART through the centre (Figure 5).

### Operational Best Practices

#### Sensitization for all hospital staff and collaborative referral system

All healthcare providers at the hospital were internally oriented to PITC, and all clinicians are trained to introduce HIV testing and its importance to every patient. Therefore, all women and children at intake points receive initial counselling from the attending clinician. Nurse counsellors report that counselling is streamlined and easier to conduct because the clinician has already introduced the topic of HIV, and hospital staff report that the sensitization training alleviated the stigma among clinicians surrounding testing and counselling.

#### Nurse counsellors serve full-time in key areas of paediatric intake

As a result of the restructured client flow pathway, all the children eligible for counselling and testing are captured, the acceptance of counselling and testing has increased, and the waiting time for HIV test and baseline results has reduced because additional visits to testing clinics are no longer required. Nurse counsellors are hired from the cohort of local retired nurses with experience in various counselling disciplines, thereby avoiding interference with current public sector staff. As qualified nurses, the nurse counsellors are able to carry out other care duties when needs arise in the outpatient clinic or on the wards. Due to the direct referrals or escorts provided by nurse counsellors, client follow-up and linkages to on-site services and support groups has also been strengthened.

#### Task shifting to peers

A group of lay counsellors called Mothers to Mothers (M2M) assist with counselling in the labour ward and the neonatal unit. M2M is a group of women living with HIV who have been trained to engage as peer counsellors for women, particularly pregnant women, to encourage them to accept counselling, testing, and HIV care. The peer counsellors also follow-up with HIV-infected women and new mothers in the community, and continue to provide peer counselling and psychosocial support on disclosure, family nutrition, and seeking HIV and other health care. This provides further evidence for the use of task shifting to peers in PITC delivery models. A similar inpatient PITC model in Malawi [20] had piloted a programme that utilized lay counsellors and volunteer “client escorts” in a large paediatric ward. These additional volunteers significantly increased the number of children offered HIV testing, reduced the time between hospital admission and testing, and maintained high testing rates. Client escorts were able to move about the ward to engage caregivers and clinicians (particularly when counsellors remained in the testing rooms), advocate testing with families, accompany them to the testing room, and provide additional counselling as required.

#### Two-way register for mother-baby pair follow-up care

The Ministry of Health in Zambia has adopted the *Sinazongwe* model for tracking mothers and HIV-exposed infants in a two-register maintained both at community and facility level [29]. In this paper-based system, antenatal and birth registers are synced between community health worker records and facility registers on a monthly basis. Thereby, there is consistency in records for follow-up care, and PMTCT peer supporters are able to follow-up with the mother-baby pair at the community level, regardless if the child was born at home or in the facility.

#### Package of on-site and local referral services for children and families

A number of local and international partnerships at LGH have facilitated an accompanying package of care and support services for children and their families. These services include psychosocial counselling for children and caregivers in the family support unit, community gardens aimed at building caregiver skills, and nutritional support for the most vulnerable families and children (e.g. ready to use therapeutic foods, food baskets). Counsellors can emphasize the availability of these services to their clients.

#### Secure antiretroviral and material supply chains

LGH began providing free antiretrovirals in 2003. Counsellors report that clients are more willing to test knowing that ART is available on-site. The hospital has maintained a consistent procurement and supply chain management system for antiretrovirals and other key supplies like test strips. From a management model, the pharmacy and laboratory managers are involved in the paediatric department’s operations and the aforementioned PITC reviews, which provided real-time consumption data to be provided in a timely way to central distribution centres.

#### Real-time data review

A full-time data clerk managed a database designed for indicators in paediatric HIV care and treatment during the course of PITC roll-out, an important tool for active management. Weekly, multidisciplinary meetings with a PITC leadership team reviewed indicators, cases of interest, defaulting clients, and any arising challenges. The weekly meetings are attended by all the staff in the paediatric department, nurse counsellors, laboratory head, pharmacist, data manager, family service unit counsellors, the maternity-in-charge, and clinical care manager. Real-time data analysis and programme monitoring provided critical feedback and immediate corrective action on PITC operations and client cases, across all key departments.

## Discussion

The hospital’s model for providing routine PITC proved feasible, and resulted in high rates of HIV counselling and testing for admitted and outpatient children. This paper seeks to elucidate these best practices to better inform policy guidelines, implementation, and scale-up of PITC in healthcare facilities. Yet despite these operational successes, there remain notable challenges to institutionalizing PITC for hospitalized children in high HIV settings.

First, a number of resource investments are required for PITC scale-up. The model relies on a secure procurement and supply chain system, and additional human resources for counselling and follow-up support. Reorganization of space and patient flows may be required to provide adequate counselling space; this has been a challenge for LGH’s surgical and OPD wards. These are considerations for larger sustainability planning at facility and management levels. Second, hospital staff sensitization is required for full acceptance of PITC as a routine, and beneficial, practice. Continued trainings and performance management is required to address issues in the provision of timely and comprehensive counselling.

Third, despite decentralized capacity for DNA PCR, there are formidable challenges to early infant diagnosis, including the turnaround for PCR results and managing loss to follow-up if families do not return for results. Additionally, increasing EID coverage requires significantly strengthened linkages to maternal and newborn care services. PMTCT counselling must emphasize infant follow-up, and health records and data management must be designed to enable timely identification of infants requiring early exposure testing. PITC services must also be better integrated into immunization and vaccination programmes to enable early identification. These challenges will become even more pronounced as EID capacity becomes further decentralized, and when LGH begins serving as a district hub for receiving DBS from, and results for, outlying facilities.

Fourth, poor involvement of male partners, particularly in a setting where men are the family decision makers, must be addressed in programming. As discussed, consent from male partners was the most common reason caregivers declined testing for their children. Robust community mobilization efforts must seek to involve the male members of family units in service access and in reducing loss to follow-up. Fifth, long-term paediatric HIV services and family support requires a package of accessible and comprehensive services; this is particularly challenging for families living in areas that are far or difficult to access from the hospital. This follow-up must target breastfeeding infants who require confirmatory testing after cessation, HIV-infected children enrolled in care, and children who have initiated ART. Efforts to maintain programme retention are likely to require community-level outreach and additional social services linked to the hospital’s quality care. Distance, poor referral networks, and stigma also lead to late referrals or service access, a significant constraint for paediatric care and clinical outcomes.

Future research should examine operational experiences with these, and other, challenges in PITC implementation for the benefit of improved practice. Paediatric PITC is still a new strategy, and managers in implementation and scale-up require clear guidance on best practices and key delivery considerations, including disclosure, age of consent for HIV tests, and adolescent care.

Finally, operational evidence from our case study hospital in Zambia emphasizes that routine PITC is particularly relevant for inpatient children in settings with generalized HIV epidemics, and should be scaled-up as a method of identifying HIV-exposed and infected infants and children that require HIV care, prophylaxis, and antiretroviral treatment. Routine PITC in the department of paediatrics at Livingstone General Hospital has resulted in nearly 100% uptake of counselling and testing for eligible children. Managers emphasize that there is a high acceptability of testing among caretakers, and no adverse social consequences have been observed or reported through civil society. The increase in the counselling and testing rate has been accompanied by an increase in the number of children enrolling in HIV care.

In conclusion, clear national and institutional policy guidance was an important catalyst for sensitizing hospital staff on PITC. PITC was quickly institutionalized by training all clinicians to offer initial counselling on HIV testing, and positioning full-time nurse counsellors in key intake areas to capture all children. The hospital’s ability to immediately provide rapid test results and collect dry blood spots for DNA PCR has been critical in reducing loss to follow-up that is otherwise an issue if children were required to move to another clinical space. Furthermore, the hospital’s efforts to integrate paediatric ART, family support care, and linkages between PITC and other prevention and support services has created a comprehensive care system for children.
