# Correction: Institutionalizing Provider-Initiated HIV Testing and Counselling for Children: An Observational Case Study from Zambia

**DOI:** 10.1371/annotation/808bf191-73bc-4b7f-a0c3-7bbf18833a21

**Published:** 2012-05-15

**Authors:** Jane N. Mutanga, Juliette Raymond, Megan S. Towle, Simon Mutembo, Robert Captain Fubisha, Frank Lule, Lulu Muhe

There was an error in the placement of Figures 4 and 5. The figures were swapped and each figure belongs with the legend of the other figure. 

